# Salivary estrone and estradiol are associated with oral microbiome profiles in aging women

**DOI:** 10.1080/20002297.2026.2690784

**Published:** 2026-06-21

**Authors:** Maria J. Rus, Maria R. Nieto, Hyo-Jung Oh, Hosuon Yoo, Victoria Areal-Quecuty, Flavio Duarte Faria, Debora Lendines-Cordero, Aurea Simon-Soro

**Affiliations:** a Department of Stomatology, University of Seville, Seville, Spain; b Department of Library & Information Science, Jeonbuk National University, Jeonju, Korea; c Department of Computer Science & Artificial Intelligence Jeonbuk National University, Jeonju, Korea; d Endodontic Section, Department of Preventive and Restorative Dentistry, School of Dentistry, São Paulo State University (UNESP), Araçatuba, SP, Brazil; e Instituto para el Estudio de la Biología de la Reproducción Humana (INEBIR), Seville, Spain; f Andalusian Center for Molecular Biology and Regenerative Medicine (CABIMER), Sevilla, Spain

**Keywords:** Oral microbiome, salivary estrogens, estrone and estradiol, midlife women, aging, niches

## Abstract

**Objectives:**

To explore whether salivary estrogens (estrone and estradiol) are associated with oral microbiome composition in aging women, and to assess the oral cavity as a potential sentinel of systemic hormonal changes during midlife.

**Materials and methods:**

Cross-sectional study including 30 women aged 40–65 years. Saliva and microbial specimens were collected from four oral ecological niches (buccal mucosa, tongue dorsum, supragingival plaque, subgingival plaque). Microbiome composition and diversity were assessed by 16S rRNA gene sequencing, ecological indices, and co-occurrence network analysis. Salivary estrone and estradiol were quantified, and associations with oral health and microbial profiles were evaluated.

**Results:**

Estrone levels declined significantly with age and were associated with hyposalivation and lower oral health scores. Estrone was linked to increased microbial diversity on the tongue dorsum and enrichment of taxa such as *Porphyromonas*. In contrast, estradiol was positively associated with commensal genera (*Streptococcus*, *Lactobacillus*) and negatively with periodontal-associated taxa (*Fusobacterium*, *Prevotella*). Co-occurrence networks revealed niche-specific microbial shifts associated with estrogen levels.

**Conclusions:**

Salivary estrogens, particularly estrone, shape oral microbial communities in aging women. The oral cavity may act as a window into systemic hormonal changes, supporting its role as a non-invasive sentinel of women’s health during midlife.

## Introduction

Fluctuations in oestrogen levels have an impact on clinical and microbial profiles in the context of women’s health and aging [1,2]. Oestrogen levels, particularly estradiol and estrone, change significantly with age [3]. Both oestrogens are found in oral body fluids such as saliva and gingival crevicular fluid, where they interact with several locations within the oral cavity, including the periodontium, oral mucosa, tongue epithelium, and salivary glands [4,5]. Clinically, oestrogens modulate alveolar bone density, gingival keratinisation, and collagen synthesis in the periodontium [6]. These hormones affect salivary flow rate and composition (including electrolyte balance, and protein concentration), mucosal maturation, inflammation, and microbial balance, playing a crucial role in maintaining oral health, especially in aging women undergoing hormonal changes.

The interaction between the microbiome and hormones is bidirectional [7–9]. Recent research shows that certain gastrointestinal microorganisms can directly metabolise hormones. These microbial interactions indicate a potential to disrupt host regulation, disturb homoeostasis, and contribute to disease development [10,11]. This dynamic relationship is influenced by factors such as aging. Microorganisms capable of metabolising sex hormones can alter hormone availability in women, impacting physiological processes [11]. This interaction suggests that microbial shifts could serve as markers of systemic endocrine changes during midlife.

The oral cavity contains distinct microbial niches, each with unique environmental conditions and microbial communities. The buccal mucosa, tongue dorsum, tooth surface, and subgingival sulcus differ in terms of tissue type, oxygen levels, nutrient availability, and mechanical forces, which influence the composition and function of their respective microbiomes [12,13]. To date, few studies have evaluated how specific salivary oestrogen profiles relate to oral microbiome diversity and composition across different ecological niches. Specifically, while estradiol has been the primary focus of hormonal research, the role of estrone, which becomes the predominant oestrogen after menopause, remains largely unexplored in the oral ecosystem. Addressing this gap is clinically relevant, as oral microbial alterations may contribute to oral health decline and reflect broader systemic processes of aging in women.

Here, we investigated the association of circulating salivary oestrogens (estradiol and estrone) with the microbial communities of four oral niches, including the buccal mucosa, tongue dorsum, tooth surface, and subgingival sulcus. By sampling multiple oral niches, we aimed to identify niche-specific microbial patterns linked to hormonal status, and to explore the potential of the oral cavity as a sentinel of systemic changes in women’s health during midlife.

## Material and methods

### Study population

This cross-sectional study comprised a cohort of 30 aging women, aged between 40 and 65 years, who attended the Faculty of Dentistry at the University of Seville for dental check-ups from January 2020 to June 2021. This study was approved by the Ethics Committee of the CEI Sevilla Sur, Hospital Universitario Virgen de Valme, Sevilla, Spain (approval number 1532-*N*-21). All participants provided written informed consent prior to enrolment.

To elucidate the hormonal impact on the oral microbial ecology of adult women, we employed a multifaceted approach by obtaining and assessing the clinical, microbial, and salivary hormonal profiles of each participant. The clinical profile included a comprehensive dental examination, along with information on age and menstrual status. The oral microbial profile was determined by collecting and sequencing samples from various oral niches, including the buccal mucosa (BM), tongue dorsum (TG), supragingival plaque from tooth surface (TH), and subgingival plaque from subgingival sulcus (GM). The hormonal profile was established by measuring salivary levels of estradiol and estrone in stimulated saliva samples. Inclusion criteria were defined as women aged between 40 and 65 years. Exclusion criteria included having fewer than six natural teeth, the presence of incapacitating diseases that would hinder the independent collection of clinical data or biological samples, ages below 40 or above 65 years, use of antibiotics or antifungals within three months prior to sample collection, or the use of mouthwashes containing oral antiseptics within one month before sampling. Additionally, women who did not provide informed consent were excluded from the study.

### Sample collection

Prior to the appointment, all participants were instructed to abstain from eating, drinking, smoking, and all oral hygiene procedures (e.g. toothbrushing, flossing, mouth rinsing) from the night before. All samples were collected in the morning between 8:00 AM and 11:00 AM to minimise the effects of diurnal variation on hormonal levels and the oral microbiome [14].

Five oral samples were collected from each participant: four samples from distinct oral niches for microbiome sequencing, and one stimulated saliva to assess salivary flow rate and oestrogen levels. The oral niches sampled were the tongue dorsum (TG), buccal mucosa (BM), tooth surface (TH), and subgingival sulcus (GM). These sites were selected to represent the distinct ecological environments of the oral cavity, following established sampling protocols for oral biogeography [12,13]. This selection allowed for a comprehensive analysis of niche-specific microbial responses by covering both shedding mucosal surfaces and stagnant biofilm sites.

For the tongue niche (TG), the centre of the dorsum was swabbed for 10 seconds using a sterile flocked nylon swab. For the buccal mucosa niche (BM), the entire area on both sides was swabbed for 10 seconds with a new, sterile swab. Each swab was immediately placed into a cryotube containing 1 mL of DNA/RNA Shield^TM^ (Zymo Research, Irvine, CA, United States). For the tooth niche (TH), supragingival plaque was collected from the central incisor, first premolar, and first molar of the maxillary right and mandibular left quadrants, excluding surfaces with fillings or caries. This selection aimed to represent the different dental groups as well as both dental arches. In cases where the selected teeth were missing or not natural, the next available tooth of the same group was sampled. Using a sterile blunt periodontal probe, the buccal and lingual surfaces were scraped in a disto-mesial direction. The collected plaque was immersed in 1 mL of DNA/RNA Shield^TM^ contained in a cryotube. For the subgingival niche (GM), subgingival plaque was collected from the same teeth using a sterile Gracey curette. The curette was carefully inserted into the gingival sulcus along the buccal and lingual surfaces in a disto-mesial direction. The collected plaque was placed into cryotubes containing 1 mL of DNA/RNA Shield^TM^.

For stimulated saliva collection, participants were provided with a piece of paraffin wax to chew. They were instructed to continuously chew the wax and drool all generated saliva into a 50 mL tube over a period of 5 minutes. All samples were kept on ice until transported to a −80 °C freezer. Clinical samples were processed within one hour to ensure the integrity of the collected biological material. Saliva samples were stored at −80 °C until hormonal quantification was performed.

To ensure the reliability of the data, we included three negative controls and one positive control in the study. The negative controls were environmental controls collected on the days of sample collection, representing the dental clinic environment and dental instruments. The positive control was a commercially available bacterial mock community, the ZymoBIOMICS^TM^ Microbial Community DNA Standard (Zymo Research, Irvine, CA, United States).

### Clinical data collection

Clinical data were collected during the same visit, immediately following the collection of all biological samples. This sequence was designed to ensure the integrity of the biological samples.

Clinical data collection involved an extraoral examination, assessment of the temporomandibular joint (TMJ), oral mucosal examination, and evaluation of dental and periodontal status using a dental examination kit with a dental mirror, caries probe, and millimetre periodontal probe.

First, adjacent tissues to the oral cavity were examined, noting any observed alterations, especially at the commissures. Next, the TMJ function was evaluated by assessing the range of motion (opening and lateral movements) and the presence of signs and symptoms during palpation and function. Lesions observed in oral structures covered by mucosa were recorded, specifying their location.

Dental status was evaluated using the dental mirror and caries probe according to the DMFT index. This involved documenting the presence of caries lesions, fillings, tooth loss, fissures, fractures, hypoplasias, abutment teeth, dental crowns, and unerupted teeth.

Periodontal status was determined using the Basic Periodontal Exam, a simple and fast method that evaluates treatment needs [15,16]. The dentition was divided into canine-limited sextants, excluding third molars. Sextants with only one tooth were excluded, and the tooth was added to the adjacent sextant. A millimetered periodontal probe was inserted between the tooth and gingiva to measure the depth of the gingival sulcus relative to the gingival margin. Six probing points were assessed on each tooth: mesial, midpoint, and distal of the buccal and palatal/lingual sides. The presence of dental calculus or other plaque retention factors was also evaluated. Each sextant was assigned the highest value obtained.

For coding purposes in the Basic Periodontal Exam, sextants that did not have pockets of 4 mm or deeper, no calculus, overhanging fillings, and no bleeding after probing were assigned Code 0. Code 1 was used for sextants that, while otherwise healthy, showed bleeding after probing. Sextants, where dental calculus or other plaque retention factors such as overhanging fillings were observed, were given Code 2. Code 3 was assigned to sextants where the maximum probing depth in one or more teeth was between 4-6 mm. Code 4 was used when one or more teeth had a probing depth greater than 6 mm. The * code was assigned to sextants where there was an attachment loss of 7 mm or more, or if furcation involvement existed.

Based on these codes, the periodontal status of participants was classified as follows: a healthy periodontium was defined as all six sextants with Code 0. Sextants with Codes 1 and 2 were considered gingivitis. The presence of more than one sextant with Code 3 determined a state of moderate periodontitis. Codes 4 and * were considered severe periodontitis.

The clinical profile of each patient was completed with a brief questionnaire collecting information on age, menopausal status, and use of hormone therapy, classified according to the international classification of menopausal stages: premenopause (regular menses), and postmenopause (menses cessation for more than 12 months).

### Salivary measurement assays

From the stimulated saliva sample collected, both salivary flow rate and pH were determined before any further processing. The salivary flow rate was measured by decantation in a calibrated test tube. A normal stimulated salivary flow rate was established at 1.5 mL/min [17]. Participants with a flow rate below this threshold were classified as having hyposalivation, while those with a flow rate above were considered normosalivated. The salivary pH was determined using a calibrated digital pH metre (Crison Basic 20).

Following these measurements, oestrogen levels, specifically salivary 17-*β* estradiol and estrone, were quantified using enzyme immunoassay kits designed for salivary analysis. Both salivary 17-*β* estradiol and estrone levels were measured following the manufacturer's protocols (Salimetrics®, State College, PA). The assays were performed in duplicate, and the average of the duplicate results was used in the analyses. Salivary estradiol and estrone values were reported in picograms per millilitre (pg/mL).

### Bacteriome analysis

DNA extraction, 16S amplification, and sequencing were performed as described in Saúco, C. et al. [18]. Briefly, microbial DNA was extracted from samples using the DNeasy PowerSoil Pro Kit (Qiagen). The V3-V4 region of the 16S rRNA gene was amplified via PCR with Herculase II fusion DNA polymerase (Agilent Technologies, Santa Clara, CA). Amplicons were purified with AMPure beads (Agencourt Bioscience, Beverly, MA), quantified with qPCR, and qualified using TapeStation D1000 ScreenTape (Agilent Technologies, Waldbronn, Germany). Sequencing was performed on the MiSeqTM platform (Illumina, San Diego, USA).

The bioinformatics analysis of the sequenced samples followed a structured workflow to ensure high-quality data processing and interpretation of the 16S rRNA gene sequences. Initially, quality control of the raw sequence data was conducted using FastQC (v0.11.9) [19]. Sequences were then filtered and trimmed with Cutadapt (v4.4) [20] to remove low-quality reads. Specifically, the forward and reverse read sequences were truncated at positions 288 and 205, respectively, due to a decrease in quality. Additionally, the first 17 bases of the 5' end of the forward reads were trimmed, and sequences with a Phred quality score below 20 were excluded. Reads shorter than 100 nucleotides were also discarded. After quality filtering, the clean reads were imported into QIIME2 (v2023.5) for microbiome analysis [21]. The sequences were demultiplexed and denoised using the DADA2 plugin (q2-dada2) [22]. This process included dereplication, chimera filtering, and merging of paired-end reads to ensure high-quality sequence data for subsequent analyses. Amplicon Sequence Variants (ASVs) were identified and differentiated during this process.

Summary statistics and visualisations were generated to assess the data quality post-processing. Phylogenetic trees were generated by aligning the ASVs using MAFFT via the q2-alignment plugin [23] and constructing a tree with FastTree2 through the q2-phylogeny plugin [24]. These phylogenetic trees were used in subsequent diversity analyses. Alpha diversity metrics, including Faith's Phylogenetic Diversity and the Simpson index, were calculated to assess microbial diversity within samples. Beta diversity was assessed using Bray-Curtis dissimilarity to evaluate the differences in microbial community structure between samples. Taxonomic classification was performed using the Human Oral Microbiome Database (HOMD v15.23) [25]. Reference sequences and taxonomy data were imported into QIIME2, and classification was conducted using the classify-consensus-vsearch method with 99% identity, providing detailed taxonomic assignments for the ASVs [26].

### Statistical analysis

The microbiome data obtained were then exported to R (v4.3.3) [27] for further statistical analysis and visualisation. This stage involved integrating the microbial data with the clinical and salivary hormonal profiles of each patient to assess the impact of hormonal levels on oral health in aging women. To ensure the accuracy of our microbial community analysis, we performed decontamination to identify and remove contaminating DNA using the decontam package (v1.22.0) [28] in R. We employed the prevalence method, which identifies contaminants based on their prevalence across samples. Negative control samples were defined, and contaminants were statistically detected and subsequently removed from the dataset to ensure high-quality, reliable microbial profiles for downstream analyses.

Alpha diversity indices (Richness, Shannon and Simpson) were calculated using the vegan package (v2.6.6.1) [29]. The Shannon index and Richness were used to assess the overall microbial diversity across niches, while the Simpson index was selected for hormonal correlations to specifically evaluate community stability and dominance. Differences in alpha diversity between niches were assessed using Wilcoxon rank sum tests [30] and associations with hormone levels were evaluated using Pearson correlation using stats package (v4.3.3). Beta diversity was assessed using Bray-Curtis dissimilarity matrices with the vegan package, and principal coordinates analysis (PCoA) was performed to visualise the clustering patterns of microbial communities. PERMANOVA tests with Benjamini-Hochberg correction [31] were employed using the ecole package (v0.9.2021) [32] to evaluate differences in community composition between niches, and the influence of salivary hormone levels was explored using Canonical Correspondence Analysis (CCA) using vegan package with significance assessed via permutation tests.

MaAsLin2 (v1.16.0) [33] was employed to identify specific microbial taxa associated with hormone levels. Fixed effects included estradiol and estrone levels, with *p*-values adjusted using the Benjamini-Hochberg method. Significant associations were visualised using heatmaps and scatter plots using ggplot2 package (v3.5.1) [34]. Indicator species analysis with the indicspecies package (v1.7.14) [35] was used to assess the overlap of microbial taxa across oral niches, visualised with UpSet plots (ComplexUpset package v1.3.3) [36,37]. Statistical significance was set at *p* < 0.05.

### Correlation measures

We utilised correlation measuring methods to uncover microbiome relationships and employed a network model to represent their interaction complexity. The primary analytical technique involved calculating correlations among various microbial genera identified from different oral niches. Typically, correlation-based methods detect significant pairwise interactions between microbiome features using correlation coefficients such as Pearson's product-moment correlation coefficient or Spearman's rank correlation coefficient [38]. However, these methods can be sensitive to spurious relationships, especially in datasets with low density and high zero-inflation [39,40]. To address these limitations, regression-based methods and probabilistic graphical models have been employed in high-dimensional studies, although these approaches require sufficient data to avoid issues such as overfitting.

We analysed a dataset with 480 microbial variables per patient, including 120 bacterial genera identified from four oral niches (BM, TG, TH, GM). Given the unique structure of this dataset, traditional probability or regression analysis was not applicable. Therefore, we employed basic correlation measures and compared the results of Pearson [41], and Spearman [42] correlation coefficients using the pandas DataFrame. Spearman's rank correlation was selected for subsequent analyses, as it is a non-parametric method that does not assume normality or linearity, making it more suitable for compositional microbiome data with small sample sizes [38]. Additionally, Spearman's results were more coherent with the ecological patterns identified in our diversity and MaAsLin2 analyses.

Network Construction and Layout Algorithms

Graph-based representations are effective for understanding community composition and exploring pairwise associations within microbiomes. The complete set of pairwise connections defines the graph’s topology, providing a comprehensive map of relationships among nodes and edges [43,44].

To focus on significant relationships, we applied a correlation threshold of 0.4, determined through exploratory data analysis. Nodes represented bacterial variables and edges represented significant correlations. Correlations with a Spearman coefficient over 0.7 were categorised as positive (blue edges) or negative (red edges). Correlations over 0.9 were noted as the most robust interactions. Network visualisation was performed using the Kamada-Kawai [45] and Spring layout [46] algorithms, implemented with the NetworkX and matplotlib libraries in Python.

## Results

### Oral health profiles in aging women

**Figure 1.**
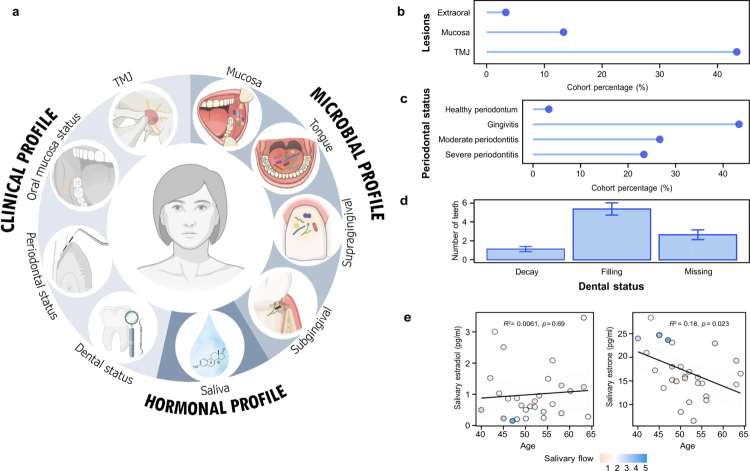
Oral health profiles in aging women. a) Schematic representation of integrated approach employed in this study, assessing clinical, oral microbial, and salivary hormonal profiles of each participant. Clinical data includes extraoral and temporomandibular joint (TMJ) evaluations, dental and periodontal examinations. Microbial profiles were determined by 16S rRNA gene sequencing samples from the buccal mucosa, tongue dorsum, supragingival plaque, and subgingival plaque. Hormonal profiles were established by measuring salivary estradiol and estrone levels in stimulated saliva. b) Bar plot illustrating the prevalence of TMJ symptoms and signs within the cohort. c) Bar plot displaying the distribution of periodontal health status among the participants, ranging from healthy periodontium to severe periodontitis. d) Bar plot showing the mean number of sound, decayed, filled, and missing teeth, reflecting the dental status of the cohort. Error bars represent the standard error of the mean. e) Scatter plots depicting the correlation between salivary oestrogen levels (estradiol and estrone) and age. Each dot represents an individual participant, coloured by their stimulated salivary flow rate, with blue normal flow (≥1.5 mL/min) and orange indicating hyposalivation (<1.5 mL/min). A linear regression line is fitted to the data, and the coefficient of determination (R²) and *p*-value are shown. The correlation between estradiol and age was not significant (r = 0.078, *p* = 0.69; Pearson), while a significant negative correlation was observed between estrone levels and age (r = −0.43, *p* = 0.023; Pearson).

By integrating clinical, microbial, and hormonal profiles, we explored a multifaceted approach to studying how oestrogen influences oral niches in aging women (Figure 1A). The study cohort included 30 women with a mean age of 51.5 years (SD ± 6.38 years). Considering the life stage of the participants, we included information on menstrual status. From our cohort, 40% consisted of premenopausal women, while 60% were postmenopausal (Table 1).

**Table 1.** Overview of the demographic, clinical, and hormonal characteristics of the cohort of aging women (*n* = 30). Data are presented as mean ± standard deviation (SD) for continuous variables and as percentages for categorical variables.

Variable	Mean ± SD/cohort %
Age (years)	51.5 ± 6.38
Menstrual status	
Premenopausal women	40%
Postmenopausal women	60%
Extraoral lesions	3.33%
Oral mucosa lesions	13.33%
TMJ status	
Symptoms	33.3%
Signs	33.3%
Periodontal status	
Healthy periodontium	3.33%
Gingivitis	43.33%
Moderate periodontitis	26.67%
Severe periodontitis	23.33%
Not evaluable	3.33%
Dental status (number of teeth)	
Sound	16.37 ± 5.56
Decay	1.13 ± 1.48
Filling	5.4 ± 3.58
Missing	2.67 ± 2.80
Saliva flow rate (mL/min)	1.43 ± 1.12
Hyposalivation	53.33%
Salivary estradiol level (pg/mL)	0.99 ± 0.85
Salivary estrone level (pg/mL)	17.16 ± 5.34

Extraoral lesions were present in 3.33% of the cohort and oral mucosa lesions were found in 13.33% of the participants. Regarding TMJ status, 33.3% of the women showed symptoms of temporomandibular joint (TMJ) problems, and another 33.3% showed clinical signs of TMJ problems (Figure 1B). Regarding periodontal status, 3.33% of the cohort had a healthy periodontium, 43.33% had gingivitis, 26.67% had moderate periodontitis, and 23.33% had severe periodontitis (Figure 1C).

For dental status, the average number of sound teeth was 16.37 (SD ± 5.56). The mean number of decayed teeth was 1.13 (SD ± 1.48), fillings were 5.4 (SD ± 3.58) and the missing teeth were 2.67 (SD ± 2.80) (Figure 1D).

We explored the relationship between salivary hormones and age (Figure 1E). We found no significant correlation between salivary estradiol levels and age (Figure 1E left plot, r = 0.078, *p* = 0.69). On the contrary, a significant negative correlation was observed between salivary estrone levels and age (Figure 1E right plot, r = −0.43, *p* = 0.023). Salivary measurements indicated that the average saliva flow rate was 1.43 mL/min (± 1.12), the mean salivary estradiol level was 0.99 pg/mL (SD ± 0.85), and the salivary estrone level was 17.16 pg/mL (SD ± 5.34). Notably, 53.33% of the women had a salivary flow rate below the normal cutoff of 1.5 mL/min, indicating hyposalivation under stimulated conditions.

### Oral biogeography: microbial variations in aging women

**Figure 2.**
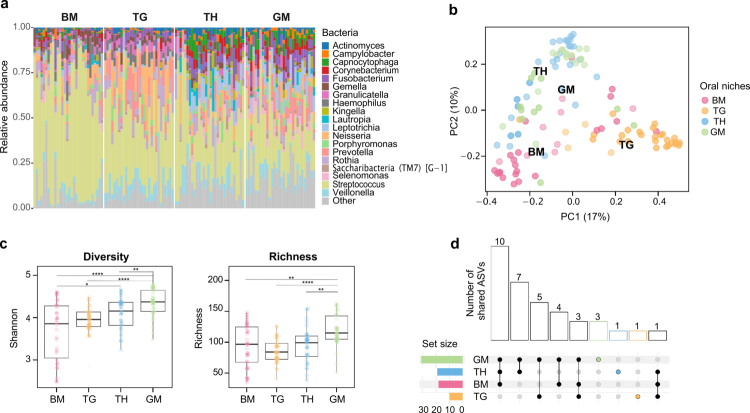
Oral biogeography: Microbial variations in aging women. a) Bar plot displaying the relative abundance of the top 20 bacterial taxa across each oral niche: buccal mucosa (BM), tongue dorsum (TG), supragingival plaque from tooth surface (TH), and subgingival plaque from subgingival sulcus (GM), with each bar representing an individual participant. b) Principal Coordinate Analysis (PCoA) plot illustrating the dissimilarity of microbial communities based on oral niche, with each dot representing an individual sample coloured by its respective niche. The closer the points, the more similar the microbial communities. Distinct clusters are observed for each niche, indicating niche-specific microbial community structures. All pairwise comparisons between niches were statistically significant (*p* < 0.05; PERMANOVA adjusted by Benjamini-Hochberg method). c) Box plots depicting microbial diversity (Shannon index) and richness across the four oral niches, with significant differences between niches indicated by asterisks (*p < 0.05, **p < 0.01, ***p < 0.001, ****p < 0.0001; Wilcoxon rank-sum test adjusted by Benjamini-Hochberg method). d) UpSet plot showing the overlap and unique microbial taxa across the four oral niches. Shared taxa are represented by connecting lines at black dots, while unique taxa are colour-coded by their specific oral niche. The size of each bar indicates the number of amplicon sequence variants (ASVs) shared by the intersection of oral niches. The size of each set represents the number of significant ASVs in each niche individually.

We assessed hormone levels and microbial abundance through salivary levels of estradiol and estrone in four oral niches such as buccal mucosa (BM), tongue dorsum (TG), supragingival plaque from tooth surface (TH), and subgingival plaque from subgingival sulcus (GM). The compositional analysis showed variations in bacterial profile between these niches in each woman (Figure 2A). The analysis of relative abundance identified representative genera such as *Actinomyces*, *Campylobacter*, and *Streptococcus* across the niches. Interestingly, we found *Neisseria* present in TG samples from some women at a mean abundance of 11% (SD ± 12%). Conversely, *Leptotrichia* was found in tooth-associated niches, particularly in GM and TH samples, with a mean abundance of 8% (SD ± 4%) and 6% (SD ± 6%), respectively. In the TG niche, we observed a significant negative correlation between the bacterial populations of *Neisseria* and *Prevotella* (r = −0.40, *p* = 0.03) (supplementary Figure S1), suggesting a possible antagonistic relationship. The results revealed complex and diverse microbial communities within each niche, with some genera being more dominant in certain areas.

The clustering of the microbial community using principal coordinate analysis (PCoA) revealed different clustering patterns for each niche (Figure 2B). The BM samples formed a separate cluster from the TG samples, indicating different microbial compositions between these niches (*p* = 1.20*10^−3^; PERMANOVA). The tooth-associated niches (TH and GM) shared microbial characteristics that resulted in a significantly reduced distance between their respective clusters (*p* = 9.00*10^−3^; PERMANOVA). These results demonstrated significant differences in the structure of niche-specific microbial communities.

Next, we investigated microbial diversity and richness in the four niches (Figure 2C). The Shannon diversity index, a measure of both species richness and evenness, revealed significant differences across the niches. Specifically, the GM exhibited the highest microbial diversity, which was significantly greater than that of all other niches (*p* < 0.05). While there was considerable variation among individual samples within each niche, the TG generally showed the lowest median diversity. The richness analysis confirmed these findings, indicating that the GM niche also had a significantly higher number of unique bacterial species compared to the other niches (*p* < 0.01). This suggests a higher variety of bacterial species in the subgingival plaque, likely due to the lack of environmental exposure and nutrient-rich niche.

We evaluated the overlap of the niche by identifying unique microbial taxa in different oral niches (GM, TH, BM, TG) in aging women (Figure 2D). We found 10 shared taxa within the BM, GM and TH niches, including species of *Bacteroidetes* (*Capnocytophaga*, *Tannerella*, *Bacteroidales*), *Proteobacteria* (*Cardiobacterium*, *Kingella*, *Lautropia*), *Firmicutes* (*Selenomonas*, *Veillonellaceae*), *Saccharibacteria* (TM7) and *Actinobacteria* (*Olsenella*). These taxa showed highly significant adjusted *p*-values (*p* < 0.05), indicating strong associations between these niches. In the GM niche, significant taxa included *Peptococcus*, *Saccharibacteria* (TM7), and *Ottowia* (all with *p* < 0.05). The TG niche was notably characterised by the presence of *Stomatobaculum* (*p* = 0.032), indicating a significant niche-specific association. The TH niche showed a strong association with *Abiotrophia* (*p* < 0.005). These findings suggested robust associations between specific microbial taxa and their respective niches, consistent with the unique landscape conditions of each site in aging women.

To further explore the environmental factors of these niches, we analysed salivary pH and its associations with salivary flow rate, age, and hormone levels. The mean salivary pH in the cohort was 7.49 (SD ± 0.63). We found a significant positive correlation between salivary pH and stimulated salivary flow rate (r = 0.433, *p* = 0.021) (supplementary Figure S2), indicating that higher flow rates were associated with a more alkaline pH. No significant correlation was observed between salivary pH and age (r = −0.27, *p* = 0.17) (supplementary Figure S3). However, we found a significant positive correlation between salivary pH and estrone levels (r = 0.507, *p* = 0.0083), although no significant association was apparent with estradiol (r = −0.231, *p* = 0.256) (supplementary Figure S4 and S5).

Furthermore, salivary pH demonstrated significant niche-specific correlations with the abundance of several bacterial genera, with the highest number of associations observed in the subgingival sulcus (GM). For instance, in the GM, higher pH was positively correlated with genera such as *Treponema* (r = 0.377, *p* = 0.047) and *Saccharibacteria* (TM7) (r = 0.541, *p* = 0.002). In the tongue dorsum (TG), *Haemophilus* showed a positive correlation with pH (r = 0.394, *p* = 0.037). Conversely, in supragingival plaque (TH), *Streptococcus* was negatively correlated with pH (r = −0.414, *p* = 0.028), whereas in the buccal mucosa (BM), *Cardiobacterium* was positively correlated (r = 0.487, *p* = 0.008). A complete list of significant correlations is available in the supplementary information. These results suggested that salivary pH is a significant environmental variable associated with hormonal status and the local, niche-specific microbial composition.

### Hormonal associations with microbial diversity in oral niches of aging women

To examine microbial communities in the context of ecosystem ecology, considering both living (microbial communities) and non-living (salivary hormonal levels) components of the oral environment, we assessed the community-level, niche-dependent, and cell-to-cell effects [47].

**Figure 3.**
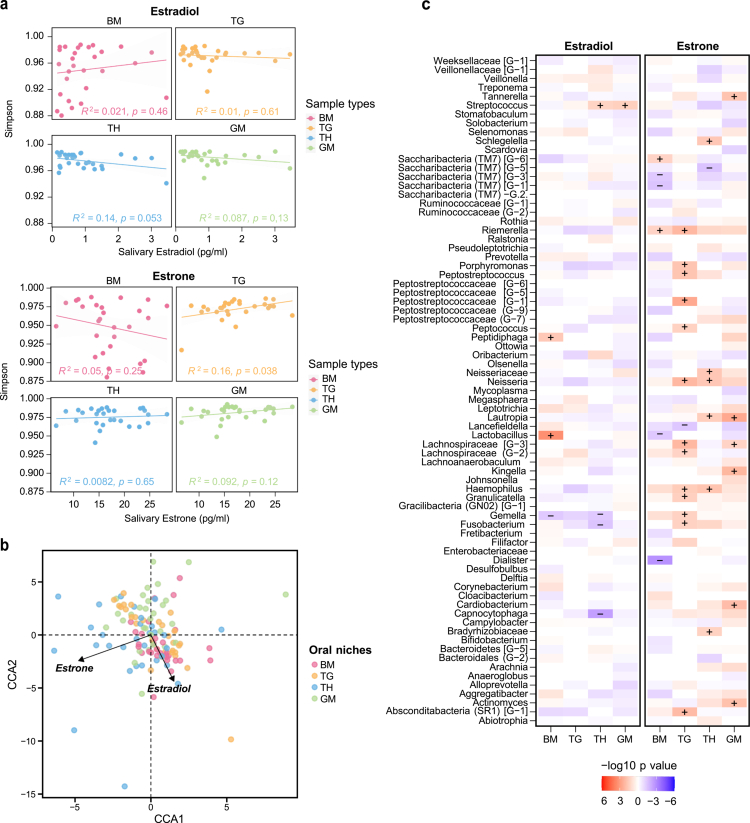
Association of salivary oestrogens with microbial diversity and community structure. a) Scatter plots depicting the correlation between salivary hormone levels (estradiol and estrone) and microbial diversity (Simpson index) in each oral niche. Linear regression lines and associated statistics (R², *p*-value) are shown for each niche. A significant positive correlation was observed between estrone levels and diversity in the TG (r = 0.39, *p* = 0.038; Pearson). b) Canonical Correspondence Analysis (CCA) biplot illustrating the association of salivary oestrogen levels (estrone and estradiol) with microbial community structure across different oral niches. Each dot represents a sample coloured by niche, and arrows indicate the direction and magnitude of the effect of each hormone. The permutation test for the CCA model was significant (*p* = 0.023; PERMANOVA adjusted by Benjamini-Hochberg method). c) Heatmap displaying associations between salivary hormone levels (estradiol and estrone) and the relative abundances of bacterial genera in different oral niches, as identified by MaAsLin2 analysis. Positive associations are shown in red, with significant associations indicated by a "+". Negative associations are shown in blue, with significant associations indicated by a "-" (*p* < 0.05; Linear model adjusted by the Benjamini-Hochberg method).

At the community level, we used Canonical Correspondence Analysis (CCA) to identify key environmental drivers, such as salivary oestrogen levels (estrone and estradiol), that were associated with the distribution and abundance of microbial taxa (Figure 3B). The permutation test for the CCA model was significant (*p* = 0.023), indicating a robust association between hormone levels and microbial community structure. The CCA plot showed that samples from different oral niches had varying associations with oestrogen gradients. The direction and length of the arrows for estrone and estradiol indicated distinct patterns of correlation with microbial community structure. Estrone showed a more pronounced association along a specific axis, while estradiol was linked to a different set of taxa along a distinct axis. This suggested that while some taxa may be negatively associated with both hormones, the primary positive associations for each hormone involved different microbial groups.

To further investigate these associations, we analysed the microbial diversity using the Simpson index and its correlation with salivary hormone levels (estradiol and estrone) (Figure 3A). Our analysis revealed a striking hormone- and niche-specific pattern. Notably, we found no significant correlation between estradiol levels and microbial diversity in any of the sampled niches (BM: r = 0.14, *p* = 0.46; TG: r = −0.1, *p* = 0.61; TH: r = −0.37, *p* = 0.053; GM: r = −0.3, *p* = 0.13). In stark contrast, a significant positive correlation was observed between salivary estrone levels and microbial diversity, an effect that was exclusively confined to the tongue dorsum (TG) niche (r = 0.39, *p* = 0.038). This association was absent in all other niches analysed (BM: r = −0.22, *p* = 0.25; TH: r = 0.09, *p* = 0.65; GM: r = 0.3, *p* = 0.12). These findings underscore a highly localised relationship, indicating that the association between estrone and microbial diversity is remarkably specific to the lingual environment.

At the niche level, we examined associations between salivary hormone levels (estradiol and estrone) and the relative abundances of various bacterial genera in different oral niches using MaAsLin2 analysis (Figure 3C). The resulting heatmap highlighted these associations, indicating which bacterial taxa were significantly correlated (*p* < 0.05) with hormone levels. For estradiol, positive associations included *Streptococcus* in the TH and GM niches and both *Peptidiphaga* and *Lactobacillus* in the BM. In contrast, significant negative correlations were found with *Gemella* in the BM and TH niches, and *Fusobacterium* and *Capnocytophaga* in the TH niche. These results suggest that estradiol is positively associated with certain commensal taxa while being negatively associated with potential pathogens across different oral niches, highlighting its potential role in the overall microbial balance in aging women.

Estrone was associated with a broader range of taxa across the various niches, a pattern most pronounced on the tongue. Elevated estrone levels were associated with increased abundances of 13 taxa, including *Riemerella*, *Porphyromonas*, *Peptostreptococcus*, *Peptostreptococcaceae* [G-1], *Peptococcus*, *Neisseria*, *Lachnospiraceae* [G-3] and [G-2], *Haemophilus*, *Granulicatella*, *Gemella*, *Fusobacterium*, and *Absconditabacteria* (SR1) [G-1]. Conversely, a negative association was noted with *Lancefieldella*.

In the BM, estrone showed positive associations with two species of *Saccharibacteria* (TM7), [G-6] and [G-1], and negative associations with two other species of the same genus, [G-3] and [G-1]. Additionally, a strong negative correlation with *Dialister* was observed. It was noteworthy that estrone and estradiol were associated with *Lactobacillus* abundance in opposite directions in the BM. High estrone levels were negatively correlated with *Lactobacillus* in the BM, whereas estradiol showed a positive correlation.

In the TH, estrone was predominantly associated with positive correlations. Elevated estrone levels correlated with higher concentrations of *Schlegelella*, *Neisseriaceae*, *Neisseria*, *Lautropia*, *Haemophilus*, and *Bradyrhizobiaceae*, with only *Saccharibacteria* (TM7) [G-5] showing a negative association. In the GM niche, exclusively positive associations were found with *Tannerella*, *Lautropia*, *Lachnospiraceae* [G-3], *Kingella*, *Cardiobacterium*, and *Actinomyces*. These findings suggested a complex relationship between oestrogen levels and microbial ecology in the oral environment of aging women, where certain bacterial genera were more prevalent depending on hormone type and levels.

**Figure 4.**
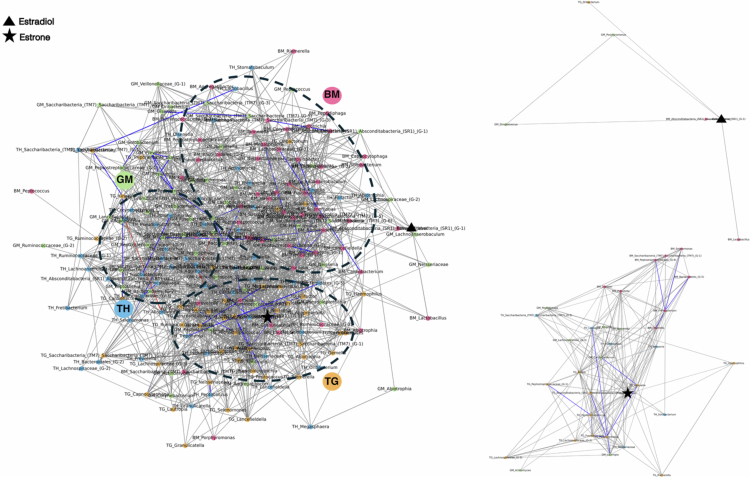
Correlation networks of oral microbial taxa and salivary oestrogens. Correlation networks depicting microbial interactions within and between oral niches (left panel), as well as correlations between estrone (upper right) and estradiol (lower right) levels and specific microbial taxa. The correlations represented are those with a Spearman's nonparametric rank correlation coefficient greater than 0.4. The distance between nodes or variables (microbial taxa and oestrogen levels) is inversely proportional to the correlation value, indicating that closer nodes are more highly correlated. Strong correlations (Spearman over 0.7) are depicted in blue, with 45 strong relationships identified.

At the cell-to-cell level, we utilised correlation measures to reveal relationships between different microbial variables from various oral niches, represented through a network model (Figure 4, left). Using Spearman's nonparametric rank correlation coefficient, we constructed a network comprising 211 oral microbiome variables, including circulating salivary estradiol and estrone levels. Each node represented a variable, with the distance between nodes being the inverse of the correlation value, indicating that closer nodes were more highly correlated. Blue lines denoted strong relationships (Spearman's correlation over 0.7), with 45 strong relationships identified out of 3,274 links.

The network revealed a complex web of correlations, predominantly within specific oral niches. BM nodes dominated the left side, TG nodes clustered on the right, and a mix of GM and TH nodes occupied the bottom. This clustering indicated that microbial interactions were more robust within the same niche, reflecting niche-specific microbial communities. The complete network results, detailing all correlations, can be found as supplementary information in the Zenodo repository (DOI: 10.5281/zenodo.12783156).

Next, we analysed the specific correlations between oestrogen levels and microbial variables. Estrone was depicted close to the TG cluster, with multiple edges extending to various microbial nodes, each representing a correlation (Figure 4, upper right). Specifically, estrone levels correlated with 32 bacteria: 11 from TG, 8 from BM, 7 from GM, and 6 from TH.

Estrone exhibited distinct correlations with various microbial taxa across different oral niches. In the GM and TG niches, estrone was predominantly associated with positive correlations. The strongest positive correlations in the GM niche included *Lautropia* (*ρ* = 0.599), *Cardiobacterium* (*ρ* = 0.547), and *Kingella* (*ρ* = 0.541). Similarly, in the TG niche, *Peptostreptococcaceae* (G-1) (*ρ* = 0.553), *Lachnospiraceae* (G-3) (*ρ* = 0.518), and *Porphyromonas* (*ρ* = 0.503) showed strong positive correlations. In the TH niche, the associations with estrone were more varied, with positive correlations observed for *Neisseria* (*ρ* = 0.469) and *Haemophilus* (*ρ* = 0.428), and a negative correlation with *Saccharibacteria* (TM7) (G-5) (*ρ* = −0.491).

Conversely, in the BM niche, estrone exhibited an exclusively negative pattern of associations. The most significant negative correlations were with *Dialister* (*ρ* = −0.652) and *Peptostreptococcaceae* (G-9) (*ρ* = −0.491). Additionally, the only bacterium negatively correlated in the TG niche was *Rothia* (*ρ* = −0.548). These findings suggest that estrone promotes a thriving bacterial community in the gingival and tongue niches while inhibiting specific bacterial populations in the buccal mucosa. This underscores its complex role as a regulatory factor in maintaining microbial balance across different oral environments.

For estradiol, the network revealed significant correlations with several microbial taxa across different oral niches (Figure 4, lower right). The strongest correlation was observed in the BM with *Lactobacillus* (*ρ* = 0.600). In the same niche, a moderate negative correlation was found with *Absconditabacteria* (*ρ* = −0.445). Within the GM niche, estradiol was linked to both positive and negative correlations. *Porphyromonas* showed a moderate negative correlation (*ρ* = −0.4048), while *Streptococcus* had a moderate positive correlation (*ρ* = 0.418). In the TG, *Oribacterium* was moderately negatively correlated with estradiol levels (*ρ* = −0.4044).

## Discussion

**Figure 5.**
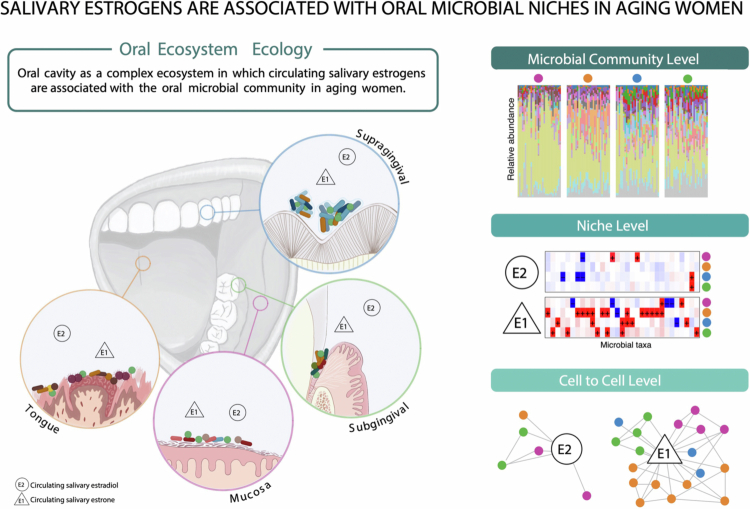
The Oral Ecosystem Ecology concept. Schematic diagram of Oral Ecosystem Ecology and the association of salivary oestrogens with microbial communities. The oral cavity is depicted as a dynamic ecosystem with distinct niches, including the tongue, mucosa, supragingival, and subgingival regions, each associated with circulating salivary estradiol (E2) and estrone (E1). The figure illustrates how these hormones are linked to microbial diversity and community structure across these niches, each with unique tissue characteristics and environmental conditions (left panel). At the *Microbial Community Level*, the relative abundance of various bacterial taxa varies across different niches, reflecting differences in microbial richness and diversity within each environment. The *Niche Level* highlights the specific associations of E2 and E1 on the microbial taxa within each niche, demonstrating niche-specific associations, with E1 having a more pronounced link as the predominant oestrogen in aging women. At the *Cell-to-Cell Level*, the network of microbial correlations shows that estrone appears as a key factor in oral ecology, correlating with the balance and interactions among different bacterial species (right panel).

The study highlights the concept of oral ecosystem ecology [48,49], as illustrated in Figure 5. This concept frames the oral cavity as a dynamic ecosystem, demonstrating how salivary oestrogen levels (estradiol and estrone) associate with microbial diversity and community structure. It considers the mouth not merely as an assemblage of tissues and bacteria but as a responsive ecosystem shaped by multiple factors, including hormonal levels. This idea is reflected through various niches within the oral cavity, such as the buccal mucosa, tongue dorsum, tooth surface, and subgingival sulcus, all of which are exposed to a shared ecosystem regulated by the salivary fluid. The multilevel approach used in this study explores these associations across three ecological layers: community, niche, and cell-to-cell level. This framework offers a comprehensive understanding of the ecological relationships within the oral microbiota.

We found that the distinct microbial compositions of different oral niches were significantly associated with specific circulating salivary oestrogens, with estradiol and estrone each having niche-specific correlations with microbial diversity. Notably, estrone showed a more pronounced association with niche-specific microbial communities compared to estradiol, and was linked to an increase in the abundance of specific bacterial taxa across various oral niches. The tongue was particularly associated with estrone levels, which correlated with its microbial composition and diversity. Thus, estrone appears to be a significant factor in intra-niche relationships, shaping the overall microbial community structure within the oral ecosystem of aging women. Understanding this concept is essential for developing targeted interventions that address hormonal status and its effects on the oral ecosystem, ultimately promoting better overall health.

Aging in women is significantly associated with hormonal variations. Fluctuations in oestrogen levels are known to modulate the development and function of oral tissues and influence the oral microbiome [50]. Oral tissues, including those of the periodontium and the salivary glands, contain oestrogen receptors, making them directly responsive to hormonal changes [51,52]. As women transition through menopause, the decline in oestrogen levels notably affects several aspects of oral health. Premenopausal stages are characterised by healthy alveolar bone maintenance, adequate mucosal keratinisation, and robust salivary flow. In contrast, the postmenopausal period involves physiological shifts such as reduced collagen synthesis, mucosal thinning, and a decrease in salivary buffering capacity [6,53]. Our results demonstrated a significant negative correlation between salivary estrone levels and age. This suggests that declining estrone levels may contribute to the deterioration of oral health observed in our cohort, particularly periodontal health.

Furthermore, over half of the study participants exhibited hyposalivation, a condition characterised by reduced salivary flow and previously linked to the impact of reduced oestrogen levels on salivary glands [53,54]. This reduced flow was, in turn, significantly correlated with a more acidic salivary pH in our cohort, a finding consistent with the known reduction of bicarbonate buffering at lower secretion rates [55]. Interestingly, we also found that higher estrone levels were directly associated with a more alkaline pH, suggesting a hormonal link to the chemical environment of the saliva. This combination of reduced salivary flow and a more acidic environment can disrupt the oral microbial balance and increase susceptibility to oral health issues, underscoring the importance of addressing these physiological changes to preserve oral health.

In our cohort, 96.67% of participants had some form of periodontal disease, with 50% presenting with moderate to severe periodontitis, a common finding in aging women with hyposalivation [56]. However, previous studies have not considered salivary oestrogen levels in these patients. This prevalence appears elevated when compared to findings in younger populations. For instance, a recent epidemiological study of adolescents and young adults reported that 59.2% of participants had some form of periodontal disease, while only 12.3% presented with periodontitis [57].

Beyond periodontal health, the dental status of our cohort was also compromised. The average number of sound teeth was relatively low, and a considerable proportion of women had decayed, filled, or missing teeth. This finding is consistent with a recent meta-analysis which demonstrated that postmenopausal women have a significantly higher DMFT index compared to younger, reproductive-age control groups (under 45 years of age) [58]. This phenomenon is attributed to the detrimental effects of oestrogen deficiency on dental health [59,60].

Furthermore, the 33.3% prevalence of self-reported TMJ symptoms in our cohort was notably high, especially when compared to the 11.3% prevalence reported in a large-scale population study of younger adults [61]. This finding supports previous evidence linking hormonal variations to TMJ health and function [62]. Collectively, these comparisons strongly suggest that the aging women in our study exhibit a poor overall oral health status. Given the observed associations between varying hormonal levels and this clinical profile, targeted interventions that consider the host's hormonal status could be essential for improving and maintaining oral health in this demographic.

Despite the findings on salivary oestrogens and their role in oral health, it is important to note that salivary oestrogen levels do not consistently correlate with those in blood. This inconsistent correlation occurs because oestrogens, which are lipophilic steroid hormones, enter the saliva primarily through passive diffusion across the acinar cells of the salivary glands. During this process, only the free and bioactive fraction, which is not bound to proteins, is able to cross these biological membranes. In the bloodstream, however, most oestrogens are bound to transport proteins, and only a small portion remains in this free and bioactive form [63–65]. Consequently, salivary oestrogen levels represent only a volatile portion of the total oestrogen pool, capturing the hormones readily available for tissue activity rather than the overall systemic levels. Moreover, the absence of robust longitudinal studies limits our understanding of the correlation between salivary and systemic oestrogen levels, especially during the menopausal transition when oestrogen levels vary significantly. Comprehensive studies are needed to elucidate this relationship, particularly regarding hormonal fluctuations in menopause.

Salivary fluid governs the oral niches by maintaining essential environmental conditions, creating a stable landscape for diverse microbial communities [66]. Circulating salivary oestrogens act as ecological selectors within the oral microbiome, favoring the growth of bacteria that thrive in oestrogen-rich environments while inhibiting others. Our results suggest that higher concentrations of estradiol are associated with a more balanced or eubiotic oral microbiota [67], a state defined by a high abundance of commensal bacteria and the suppression of potential pathogens. The protective role of estradiol, the predominant oestrogen during the fertile phase of women, is well-documented in supporting overall health [68]. This illustrates that the mouth can be considered a window to the overall condition of the body.

Specifically, we found that estradiol levels positively associate with resident bacteria like *Streptococcus* in tooth-associated niches (supra- and subgingival plaque). A similar positive association was observed for *Lactobacillus* in the buccal mucosa. *Lactobacillus* and *Streptococcus* species play dual roles in the oral microbiome, acting as both commensals and potential pathogens depending on the environmental context and specific species present. Beneficially, *Lactobacillus* species, such as *L. casei*, produce lactic acid and antimicrobial substances that inhibit cariogenic bacteria like *Streptococcus mutans* and *Porphyromonas gingivalis*, promoting a healthy oral environment [69]. *Streptococcus* species, including commensal strains like *S. mitis* and *S. salivarius*, support oral health by modulating immune responses and preventing pathogen colonisation [70].

However, this balance can shift under certain conditions. In cases of hyposalivation, common in aging populations, *Lactobacillus* species may proliferate excessively, creating an acidic environment that promotes enamel demineralisation and dental caries. Similarly, pathogenic *Streptococcus* strains, such as *S. mutans*, form biofilms and produce acids that may lead to caries [71]. The balance between these beneficial and pathogenic roles underscores the delicate equilibrium within the oral microbiome, where factors like salivary oestrogen levels can selectively promote or inhibit different microbial populations, tipping the balance toward health or disease.

Conversely, we found that the impact of estrone on the oral microbiome was more varied in terms of microbiome stability and maintenance of oral health. Higher levels of estrone were associated with potentially pathogenic or opportunistic bacteria such as *Porphyromonas* or *Cardiobacterium*, while correlating with lower abundances of resident bacteria that contribute to microbial balance. This suggests that, in aging women where estrone becomes the predominant oestrogen, there may be an imbalance in the oral microbiome. This imbalance could lead to increased susceptibility to oral health issues, as observed in our cohort.

Additionally, estrone positively correlated with microbial diversity in the tongue. ERβ is the predominant oestrogen receptor subtype in the oral epithelium, including the tongue, and may influence local microbiota through hormone-mediated changes in the epithelial environment [72]. The specific distribution and density of ERβ in the tongue compared to other oral niches may contribute to the observed microbiome variability. Thus, this finding implies that estrone could create a favourable ecological condition that promotes microbial diversity [11,73]. Broader implications of hormone-microbiome interactions extend beyond the oral cavity, as hormonal fluctuations can significantly impact the composition of the gut microbiome [74]. Moreover, other research demonstrated that oestrogen levels are correlated with increased microbial diversity in the vaginal microbiome, suggesting a consistent pattern of oestrogen-promoting microbial diversity across different body sites [75].

Network analysis revealed complex intra-niche microbial relationships, providing insights beyond the pairwise associations typically identified through traditional methods like correlation or regression analysis. Unlike these methods, network analysis explores the interdependencies within microbial communities [38], highlighting how different microbial taxa are correlated with each other and with salivary hormones like estradiol and estrone. Our study showed that estradiol and estrone were associated with microbiota differently, with estrone being linked to a broader range of microorganisms. The associations with estrone varied significantly across different niches, with no single microorganism being consistently correlated across all niches. Instead, various microorganisms showed different patterns of association depending on the niche, reflecting the niche-specific relationships with salivary hormones. This underscores the value of network analysis in understanding the complex and dynamic nature of the oral microbiota, where interconnections are more crucial than the specific types of microorganisms present.

Beyond direct hormonal associations, our findings highlight salivary pH as another key environmental factor associated with the oral ecosystem. We identified a potential physiological pathway where the host's oestrogen status, particularly estrone levels, is linked to salivary function. This was evidenced by the significant positive correlations we found between estrone and salivary pH, and between salivary flow rate and pH. This suggests a possible axis where hormonal signals modulate glandular function, which in turn alters the chemical environment of the saliva. Such an altered pH can then act as a powerful selective pressure on the resident microbiota, contributing to the niche-specific patterns we observed.

These pH-driven microbial signatures could have direct clinical implications for our aging cohort. For instance, an alkaline salivary environment, which correlated in our study with a higher abundance of proteolytic periopathogens like *Treponema* in the subgingival niche, could be a contributing factor to the high prevalence of periodontal disease we observed [76]. Conversely, reduced salivary flow, a common issue in our cohort, was associated with a lower pH that could favour acid-tolerant streptococci, increasing caries risk [77,78]. Therefore, our study highlights that salivary pH is a critical, potentially modifiable factor. Interventions aimed at maintaining a neutral pH could help steer the oral ecosystem towards a more balanced state.

Notably, this study is among the first to document simultaneous correlations between estrone, salivary flow, pH, and niche-specific microbiota in aging women, providing a more complete and integrated model of oral health. Building on this model, these results suggest that dental practitioners should consider hormonal shifts as a relevant factor in oral health assessments for aging women. Practitioners should be aware that the hormonal transition, particularly the decline in estrone, is linked to hyposalivation and an increased susceptibility to oral diseases. Consequently, we recommend a personalised preventive approach, including adjusting the frequency of dental visits and enhancing clinical monitoring for postmenopausal patients. Proactive management, such as tailored oral hygiene education and the monitoring of salivary flow and pH, could help mitigate the oral health decline associated with hormonal shifts during the aging process. Future longitudinal studies that measure these variables concurrently are essential to confirm the causal relationships proposed here and to develop targeted, oral-ecosystem-modulating therapies for aging women.

Despite the insightful findings, our study has several limitations. The cohort size of 30 participants with four distinctive oral niches provided preliminary insights but limited the generalisability of our results. Therefore, larger cohorts are necessary to confirm these findings and better represent the variability in oral microbial communities and hormonal profiles among aging women. Similar studies have emphasised the importance of larger sample sizes for more robust conclusions [79,80]. While we observed associations between hormonal levels and microbial diversity, longitudinal studies are needed to understand how these interactions evolve. Other studies have demonstrated significant shifts in the microbiome correlating with metabolic diseases, highlighting the need for temporal data [81].

Furthermore, the mechanistic pathways underlying the associations between oestrogens and microbial communities remain unclear. Although we identified correlations, the specific biological mechanisms driving these associations were not explored. Recent work has begun to uncover these pathways, suggesting that commensal bacteria can influence hormone levels and potentially impact cancer development [82]. Future research should incorporate experimental manipulation of hormone levels and advanced molecular techniques to elucidate these mechanisms.

The findings of this study provide a foundation for further exploration of the endocrine-microbiome axis. Integrating other sex hormones that shift during aging, such as progesterone and androgens, would provide a more comprehensive understanding of the complex interactions shaping the oral ecosystem. These hormones have known receptors in oral tissues and likely contribute to microbial stability. Such a multi-hormonal approach could help elucidate potential synergistic effects on oral health in aging women. Addressing these limitations in subsequent research will be critical for advancing our understanding of the role of oestrogens in shaping the oral microbiome and for developing targeted interventions to promote oral health in aging women.

## Conclusion

This study shows that salivary oestrogens, particularly estrone, decline with age and are associated with hyposalivation, poorer oral health, and site-specific shifts in oral microbiome composition. Estrone enhanced microbial diversity on the tongue, while estradiol favoured commensals and reduced periodontal-associated taxa. These findings suggest that hormonal changes during midlife influence the oral microbiome in a niche-specific manner and support the use of the oral cavity as a window into systemic endocrine transitions in women’s health.

## Supplementary Material

Figure_S4.png Figure_S4.png

Figure_S3.png Figure_S3.png

Figure_S1.png Figure_S1.png

pH_bacterial_abundance_spearman_corr.xlsx pH_bacterial_abundance_spearman_corr.xlsx

Figure_S5.png Figure_S5.png

Figure_S2.png Figure_S2.png

## Data Availability

The 16S rRNA sequencing data have been deposited in the Sequence Read Archive (SRA) of the National Centre for Biotechnology Information (NCBI) under the accession number PRJNA1137150. Clinical data and hormone profiles of each patient are included along with the sample sequencing data. The underlying code for this study is available in Zenodo and can be accessed via this DOI: 10.5281/zenodo.12783156.
