# Supplementary figures and images for: Salivary estrone and estradiol are associated with oral microbiome profiles in aging women
Source: J Oral Microbiol. 2026 Jun 21;18(1):2690784. doi: 10.1080/20002297.2026.2690784 (PMC13288545; doi:10.1080/20002297.2026.2690784)

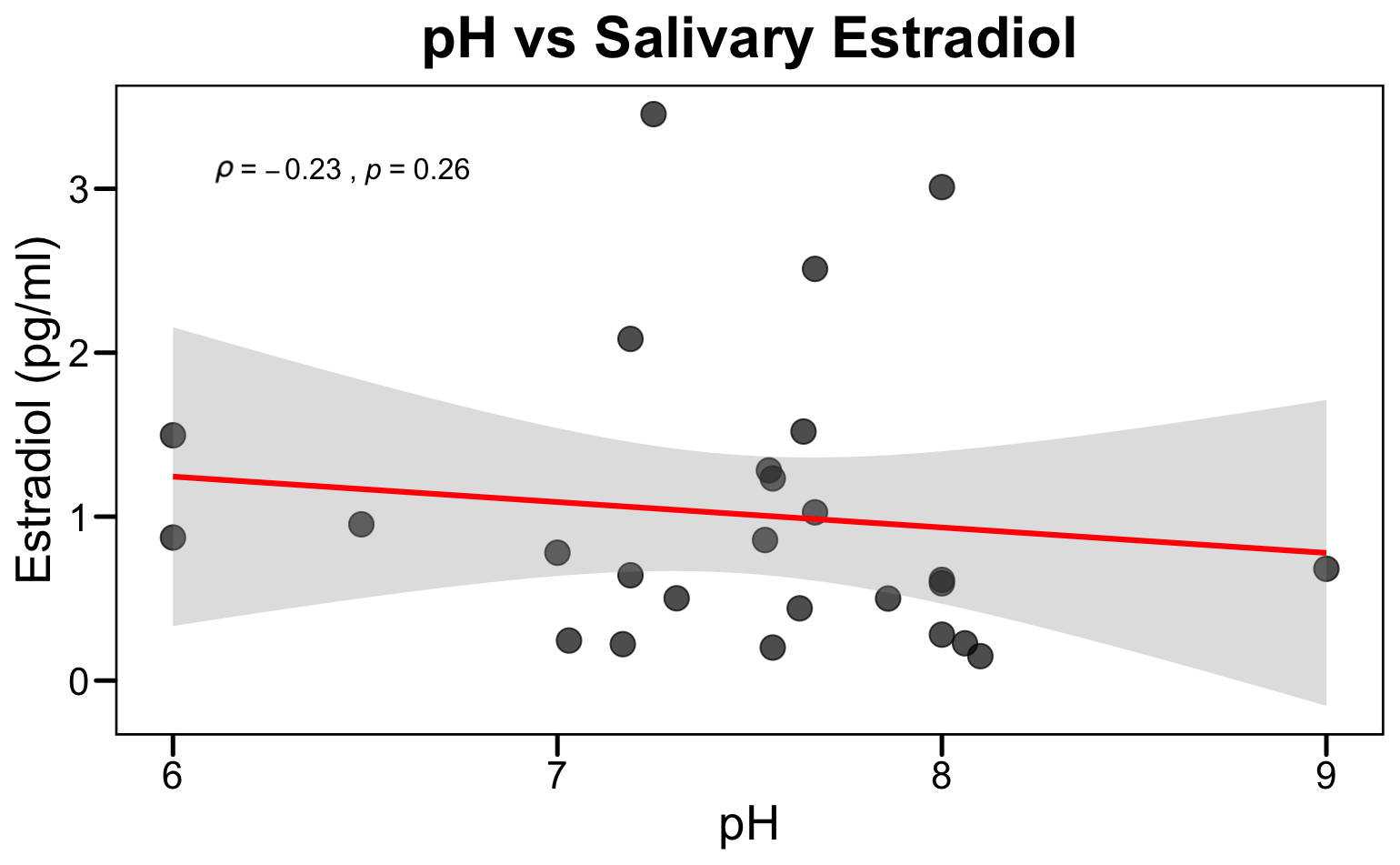

Supplement: Figure_S4.png [file ZJOM_A_2690784_SM4608.png]

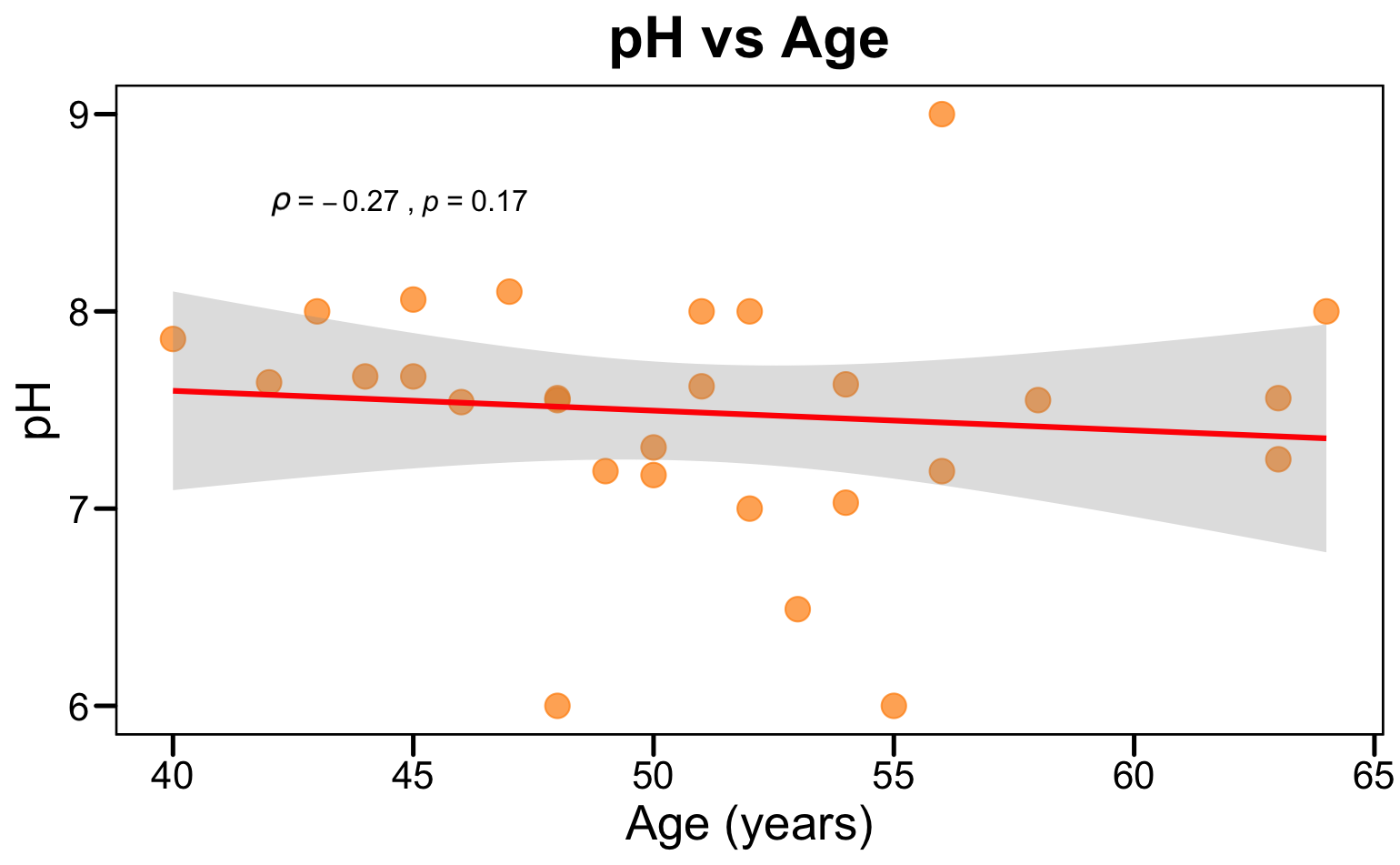

Supplement: Figure_S3.png [file ZJOM_A_2690784_SM4610.png]

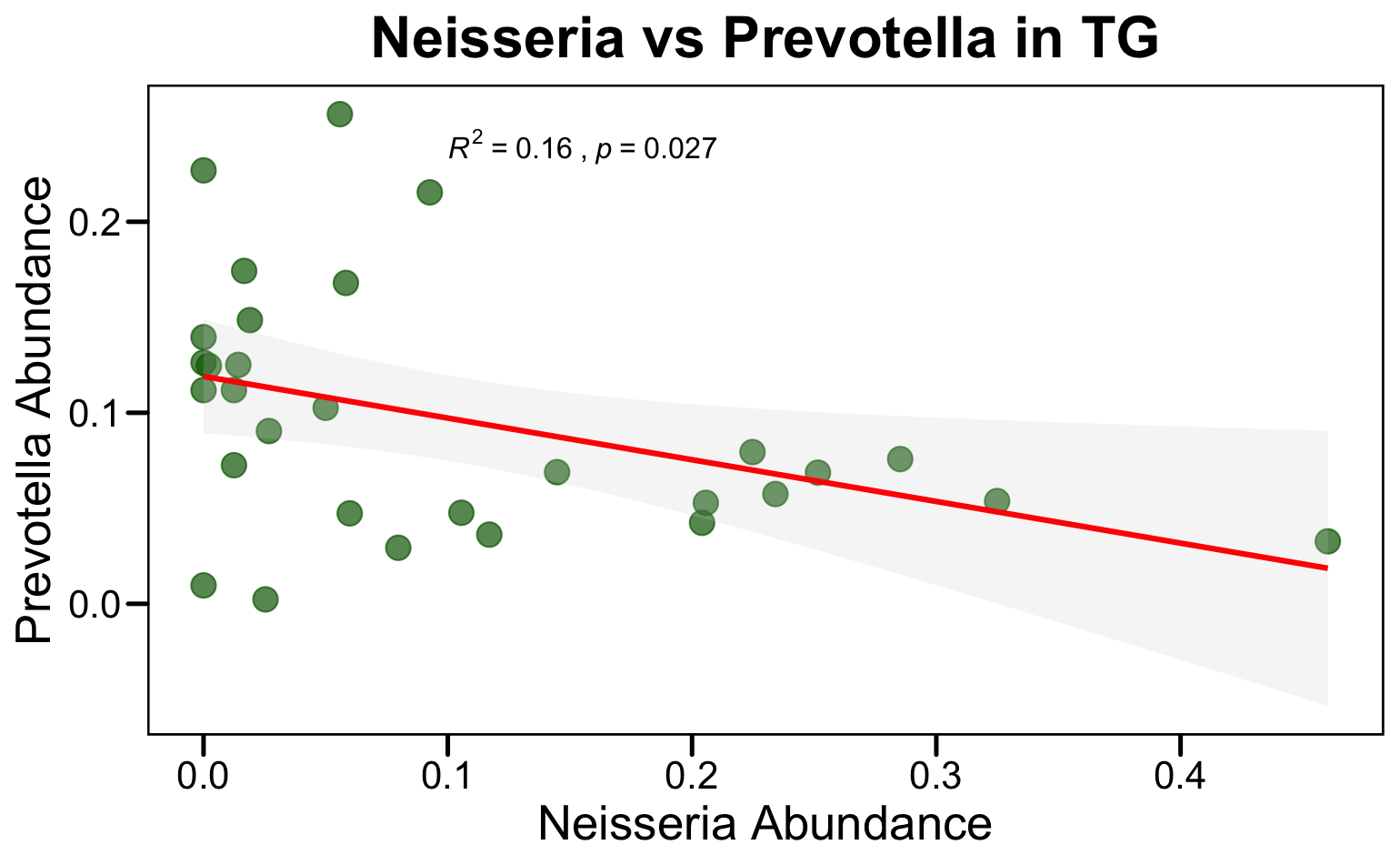

Supplement: Figure_S1.png [file ZJOM_A_2690784_SM4611.png]

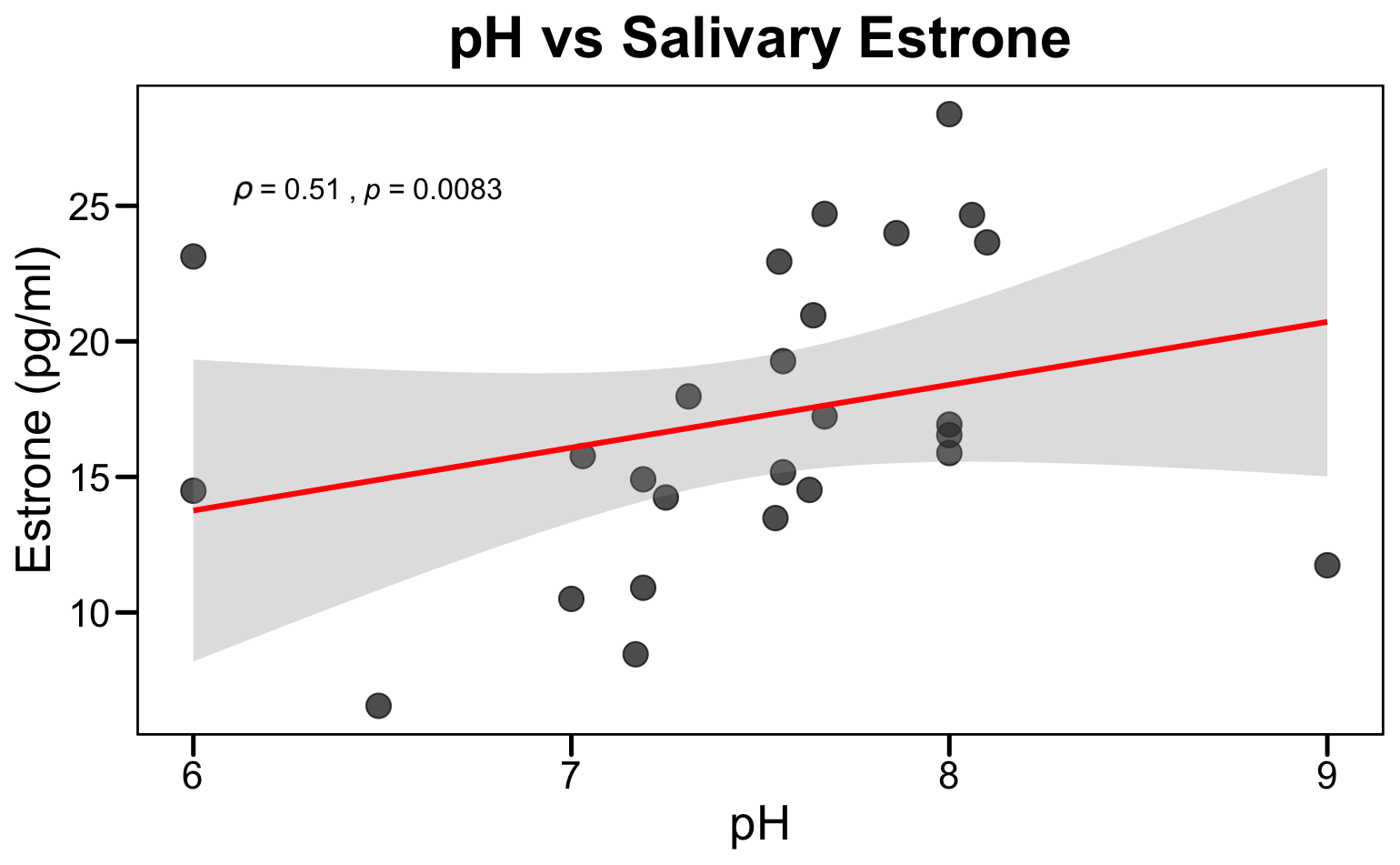

Supplement: Figure_S5.png [file ZJOM_A_2690784_SM4613.png]

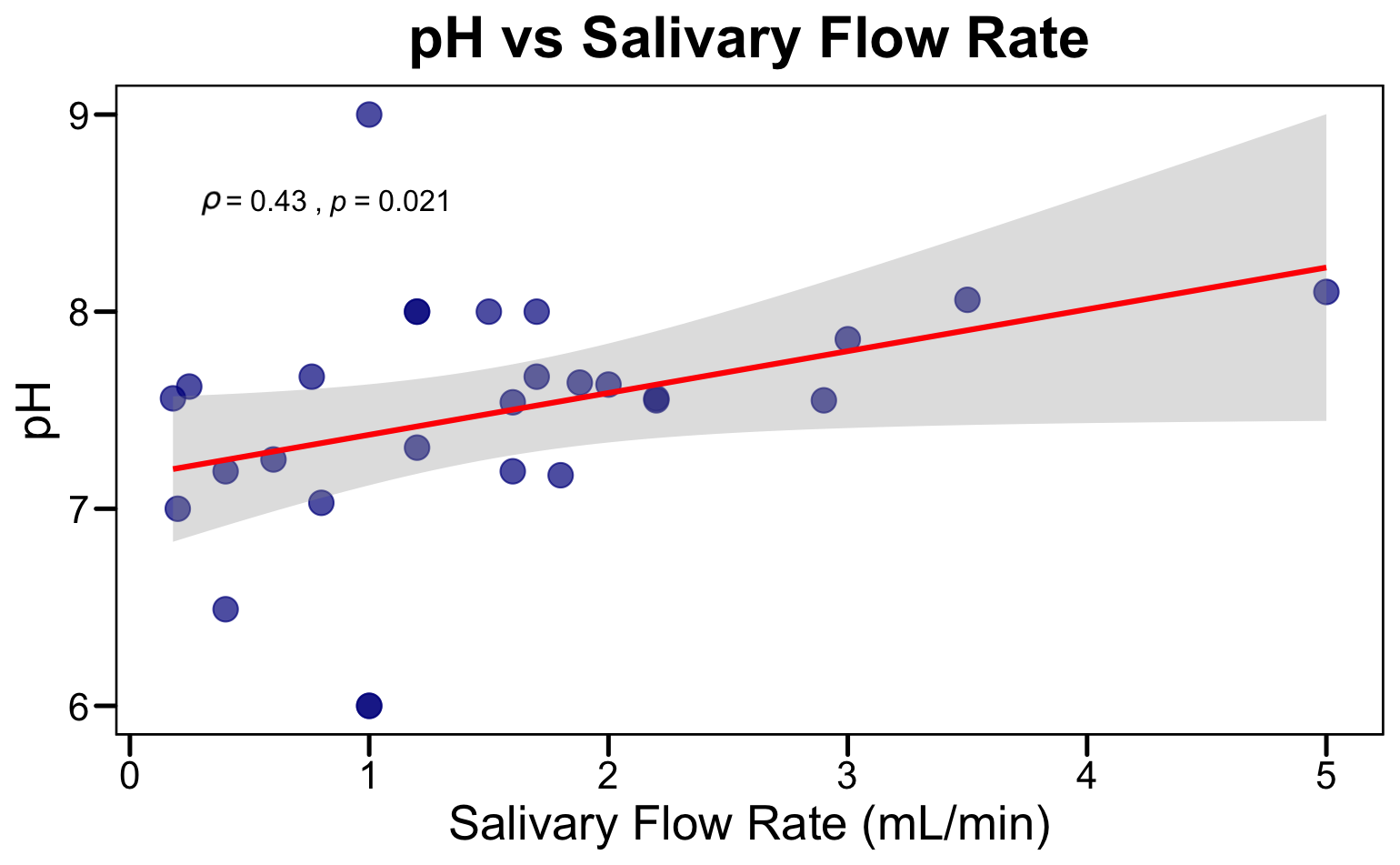

Supplement: Figure_S2.png [file ZJOM_A_2690784_SM4614.png]
